# Age-related trends in amyloid positivity in Parkinson’s disease without dementia

**DOI:** 10.18632/aging.206297

**Published:** 2025-08-06

**Authors:** Keiko Hatano, Masashi Kameyama, Masanori Kurihara, Kensuke Ohse, Ryoji Goto, Ryoko Ihara, Mana Higashihara, Renpei Sengoku, Yasushi Nishina, Kazutomi Kanemaru, Yuko Saito, Shigeo Murayama, Atsushi Iwata

**Affiliations:** 1 Department of Neurology, Tokyo Metropolitan Institute for Geriatrics and Gerontology, Tokyo, Japan; 2 AI and Theoretical Image Processing, Research Team for Neuroimaging, Tokyo Metropolitan Institute for Geriatrics and Gerontology, Tokyo, Japan; 3 Integrated Research Initiative for Living Well with Dementia, Tokyo Metropolitan Institute for Geriatrics and Gerontology, Tokyo, Japan; 4 Department of Neurology, The University of Tokyo Hospital, Tokyo, Japan; 5 Department of Neurology, The Jikei University School of Medicine, Tokyo, Japan; 6 Department of Neuropathology (The Brain Bank for Aging Research), Tokyo Metropolitan Institute for Geriatrics and Gerontology, Tokyo, Japan; 7 Brain Bank for Neurodevelopmental, Neurological and Psychiatric Disorders, United Graduate School of Child Development, Osaka University, Osaka, Japan

**Keywords:** amyloid positivity, Parkinson's disease without dementia, cerebrospinal fluid Aβ42

## Abstract

Amyloid-beta (Aβ) plays a pivotal role in cognitive decline in Parkinson’s disease (PD). The prevalence of amyloid positivity, evaluated using the cerebrospinal fluid (CSF) of patients with PD without dementia in their sixties, is lower than that in individuals with normal cognition without PD diagnosis in the same age range. However, it is unclear whether this is also the case in patients with PD without dementia in their eighties. Eighty-nine patients with PD without dementia were retrospectively classified into two groups with a cut-off age of 73 years at diagnosis: a HIGH group and a LOW group, with mean age at diagnosis of 80.2 and 64.9 years, respectively. The prevalence of amyloid positivity was significantly higher in the HIGH (30.6%) than in the LOW group (10.0%) (*p* = 0.02). The prevalence of amyloid positivity in both groups was lower than that in participants with normal cognition in the same age range. Our findings may be attributed to the shorter preclinical stage of asymptomatic cerebral Aβ deposition in PD, resulting from Aβ accelerating the transition from the asymptomatic to dementia stage. We believe that our findings will incentivize further studies to identify the best disease-modifying therapy for early PD without dementia.

## INTRODUCTION

Amyloid-beta plays a pivotal role in cognitive decline in Lewy body (LB) disease. Neuropathological studies have shown that the amyloid burden in the central nervous system is more strongly correlated with the development of dementia than α-synuclein pathology both in Parkinson’s disease (PD) with dementia (PDD) and dementia with Lewy bodies (DLB) [1–3]. Marked amyloid deposition in patients with PDD is associated with a rapid rate of cognitive decline [4]. Aside from amyloid-beta, multiple proteinopathies, including tau [5, 6], α-synuclein [7], TAR-DNA-binding protein 43 (TDP-43) [8], and vasculopathy [9] have been implicated in the pathogenesis of cognitive impairment in Lewy body disease (LBD). However, in an era when anti- amyloid-beta antibodies have been implemented as disease-modifying therapies for Alzheimer’s disease (AD), amyloid-beta remains a crucial protein of interest. The amyloid-beta concentration of cerebrospinal fluid (CSF) has robust prognostic value for cognitive decline in both PD [10–12] and DLB [13]. Thus, CSF profiles are valuable in evaluating amyloid positivity and may also be useful in selecting patients with PD who are eligible for early disease-modifying therapy.

The prevalence of PD increases approximately 10-fold between the ages of 50 and 80 years [14]. The number of patients who will be diagnosed with PD in their eighties is predicted to increase in our aging society. Previous clinicopathological studies have shown rapid progression in motor symptoms [15, 16] and earlier dementia development in patients with PD onset ≥ 80 years than that in patients with earlier PD onset [15]. However, antemortem amyloid positivity in patients with PD without dementia in their eighties at diagnosis has not yet been reported.

The prevalence of amyloid positivity in patients with PD without dementia is lower among patients in their sixties than individuals with normal cognition and no diagnosis of PD (hereinafter referred to as individuals with normal cognition) in the same age range [17, 18]. However, it remains unclear whether this is also the case in patients with PD without dementia in their eighties.

We aimed to determine the prevalence of amyloid positivity using CSF profiles in patients with PD without dementia who were in their eighties at diagnosis by applying the CSF AD biomarkers to the AT(N) classification [19].

## RESULTS

The patients' characteristics are shown in Table 1. The percentage of patients with normal cognition was 91.8% and 95.0% in the HIGH and LOW groups, respectively. All apolipoprotein E (ApoE) ε4 carriers had one ε4 allele. Quantile-quantile plots of Aβ42 levels as a representative of approximately normally distributed data are presented in Supplementary Figure 1.

**Table 1:** Clinical characteristics of the patients.

	**HIGH group (N = 49)**	**LOW group (N = 40)**	***p-* value**	**Missing data, n (%)**		test*
				**HIGH group**	**LOW group**	
Age at diagnosis and LP (y)	80.2 (± 4.4)	64.9 (± 7.3)		0	0	
Age at onset (y)	79.0 (± 4.6)	63.4 (± 7.6)		0	0	
Disease duration at diagnosis (m)	18.1 (± 13.9)	21.0 (± 15.0)	0.40	0	0	a
The interval between ^123^I-ioflupane SPECT and LP (m)	2.6 (± 9.4)	4.8 (± 10.7)	0.33	0	0	a
Male, n (%)	21 (42.9%)	18 (45.0%)	1.00	0	0	b
ApoE ε4 allele carriers, n (%)	10 (21.7%)	5 (14.3%)	0.57	3 (6.1%)	5 (12.5%)	b
Motor symptoms						
Hoehn-Yahr score	2.4 (± 0.7)	2.4 (± 0.8)	0.64	0	0	a
bradykinesia, n (%)	49 (100.0%)	40 (100.0%)	1.00	0	0	b
rigidity, n (%)	44 (89.8%)	38 (95.0%)	0.45	0	0	b
resting tremor, n (%)	24 (49.0%)	21 (52.5%)	0.83	0	0	b
recurrent falls, n (%)	8 (16.3%)	8 (20.0%)	0.78	0	0	b
gait freezing or festination, n (%)	10.0 (20.4%)	6 (15.0%)	0.59	0	0	b
Non-motor symptoms						
Presence of at least one non-motor symptom, n (%)	38 (77.6%)	34 (85.0%)	0.43	0	0	b
Autonomic nervous function						
Orthostatic hypotension, n (%)	19 (42.2%)	19 (50.0%)	0.38	4 (8.2%)	4 (10.0%)	b
^123^I-MIBG myocardial scintigraphy positivity, n (%)	28 (59.6%)	22 (59.5%)	1.00	2 (4.1%)	3 (7.5%)	b
The interval between ^123^I- MIBG myocardial scintigraphy and LP (m)	2.8 (± 12.1)	1.9 (± 6.1)	0.58	2 (9.1%)	3 (6.1%)	a
Psychiatric symptoms						
GDS scores >=6, n (%)	9 (22.0%)	10 (37.0%)	0.27	8 (16.3%)	13 (32.5%)	b
Cognitive function-related data						
Education (y)	11.8 (± 2.8)	14.1 (± 3.2)	0.003**	13 (26.5%)	12 (30.0%)	a
MMSE scores	27.1 (± 2.0)	28.3 (± 1.8)	0.014*	8 (16.3%)	8 (20.0%)	a
Normal cognition, n (%)	45 (91.8%)	38 (95.0%)	0.69	0	0	b
Comorbidity						
Diabetes mellitus, n (%)	4 (8.2%)	3 (7.5%)	1.00	0	0	b

HIGH group = patients aged ≥ 73 at diagnosis, LOW group = patients aged < 73 at diagnosis, LP = lumbar puncture, y = years, m = months, SPECT = single-photon emission computerized tomography, MIBG = meta-iodobenzyl-guanidine myocardial, GDS = geriatric depression scale, MMSE = mini-mental state examination, MCI = mild cognitive impairment, ApoE = Apolipoprotein E, ** = *p* < 0.01, * = *p* < 0.05. *P-*values represent the result of Welch’s t-test or Fisher’s exact test. *test a = Welch’s t-test, b = Fisher’s exact test.

No significant difference was observed in any clinical or imaging parameter between the two groups, except the Mini-Mental State Examination (MMSE) scores and education years. The MMSE scores were significantly lower in the HIGH than in the LOW group (27.1 ± 2.0 vs. 28.3 ± 1.8, *p* = 0.01). Years of education were significantly lower in the HIGH than in the LOW group (11.8 ± 2.8 vs. 14.1 ± 3.2, *p* = 0.003). To evaluate the relationship between aging and education years or MMSE scores in each AT(N) category, we conducted Welch’s t-test comparing the HIGH vs. LOW group for education years and MMSE scores within each AT(N) category in Supplementary Table 1A. No significant differences were observed between the HIGH and LOW group in the AD continuum category for both education years and MMSE scores.

**Figure 1:**
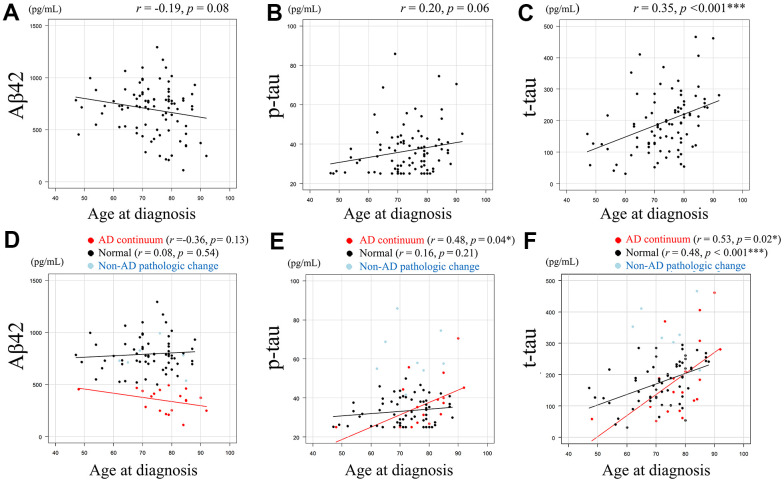
**Relationship between Aβ42, p-tau, or t-tau levels and age at diagnosis.** Scatter plots demonstrate the relationship between age at diagnosis and CSF biomarkers. Least squares regression lines are included where Pearson’s correlation analysis was performed. A negative correlative tendency (*r* = -0.19, *p* = 0.08) was observed between CSF Aβ42 levels (pg/mL) and age at diagnosis. (**A**) A positive correlative tendency (*r* = 0.20, *p* = 0.06) was observed between CSF p-tau levels (pg/mL) and age at diagnosis. (**B**) Significant positive correlation was observed between CSF t-tau levels (pg/mL) and age at diagnosis (*r* = 0.35, *p* < 0.001). (**C**) The association between age at diagnosis and each AT(N) category for CSF Aβ42, p-tau, and t-tau are shown. (**D**–**F**), with Pearson’s correlation coefficient not calculated for the non-AD category due to the small number of cases. CSF Aβ42 and age at diagnosis showed negative correlative tendency (*r* =-0.36, *p*= 0.13) in the AD continuum category, whereas this tendency was not evident in the normal category (*r* = 0.08, *p* = 0.54). (**D**) CSF p-tau and age at diagnosis showed a significant positive correlation in the AD continuum category (*r* = 0.48, *p* = 0.04), but showed no obvious tendency in the normal category. (**E**) CSF t-tau and age at diagnosis showed a significant positive correlation in both patients in AD continuum category (*r* = 0.53, *p* = 0.02) and normal category (*r* = 0.48, *p* < 0.001). (**F**) Data in AD continuum category are plotted in red, normal category in black, and non-AD pathologic change category in light blue (**D**–**F**). Aβ42 = amyloid-beta 42, p-tau = phosphorylated tau, t-tau = total tau, * = *p* < 0.05, *** = *p* < 0.001. *r* and *p* represent Pearson’s correlation and significance, respectively.

CSF amyloid-beta 42 (Aβ42) levels (pg/mL) and age at diagnosis showed a negative correlative tendency (*r* = -0.19, *p* = 0.08) (Figure 1A), and CSF tau phosphorylated at threonine 181 (p-tau) levels (pg/mL) and age at diagnosis (Figure 1B) showed a positive correlative tendency (*r* = 0.20, *p* = 0.06). Conversely, CSF total-tau (t-tau) levels (pg/mL) and age at diagnosis were significantly positively correlated (*r* = 0.35, *p* < 0.001) (Figure 1C). The association between age at diagnosis and each AT(N) category for CSF Aβ42, p-tau, and t-tau is shown in Figure 1D–1F, respectively. Pearson’s correlation coefficient was not calculated for the non-Alzheimer’s (non-AD) pathologic change category due to the small number of cases. CSF Aβ42 and age at diagnosis showed negative correlative tendency (*r* =-0.36, *p* = 0.13) in the AD continuum category, whereas this tendency was not evident in the normal category (*r* = 0.08, *p* = 0.54) (Figure 1D). CSF p-tau and age at diagnosis showed a significant positive correlation in the AD continuum category (*r* = 0.48, *p* = 0.04), but showed no obvious tendency in the normal category (Figure 1E). CSF t-tau and age at diagnosis showed a significant positive correlation in both patients in AD continuum category (*r* = 0.53, *p* = 0.02) and normal category (*r* = 0.48, *p* < 0.001) (Figure 1F).

The prevalence of amyloid positivity, that is, AD continuum, in both groups is shown in Table 2. The prevalence of amyloid positivity was significantly higher in the HIGH (30.6%) than in the LOW group (10.0%) (95% confidence interval, 1.10–17.8; odds ratio, 3.91; and *p* = 0.02), as determined by Fisher’s exact test.

**Table 2:** Comparison of the prevalence of amyloid positivity between the two groups.

	**HIGH group (N = 49)**	**LOW group (N = 40)**	**Odds ratio**	**95%CI**	***p*-value**
Amyloid positivity, n (%)	15 (30.6%)	4 (10.0%)	3.91	1.10–17.8	0.02*

HIGH group = patients aged ≥ 73 at diagnosis, LOW group = patients aged < 73 at diagnosis, CI = confidence interval, * = *p* < 0.05. *P-*values represent the result of Fisher’s exact test.

The clinical data of each AT(N) biomarker category are shown in Supplementary Table 1A. Age at diagnosis, disease duration (months), education years, MMSE scores, mild cognitive impairment (MCI) prevalence, and ApoE ε4 allele did not differ significantly in any pairwise comparisons of the three categories in each group. Specifically, to evaluate the relationship between the numbers of MCI and AT(N) categories, we conducted Fisher’s exact tests using Supplementary Table 1B for the HIGH group and Supplementary Table 1C for the LOW group, yielding *p*-values of 1.00 and 0.36, respectively. Of the six patients with MCI, the AT(N) profiles of two MCI patients who were not in the normal category were as follows: A+T-(N)- in the patient in the AD continuum category in the HIGH group and A-T-(N)+ in the other patient in the non-AD pathologic change category in the LOW group. Among the six MCI patients, the normal category in the HIGH group included one ApoE ε4 allele carrier.

## DISCUSSION

The prevalence of amyloid positivity in the HIGH group was 30.6%, which was significantly higher than the 10.0% in the LOW group (Table 2). To the best of our knowledge, this is the first study to demonstrate the prevalence of amyloid positivity using the CSF profiles of patients with early PD without dementia with a mean age of 80.2 years at diagnosis.

Regarding the clinical characteristics of the patients in the two groups (Table 1) including positivity in ^123^I- MIBG scintigraphy, we inferred that PD severity did not differ significantly between the two groups. This allowed us to observe age-related differences in the prevalence of amyloid positivity in PD. The significantly lower MMSE scores in the HIGH group compared to the LOW group may have been affected by age and the significant difference in years of education between the two groups [20], though there were missing data on education years.

In previous studies, the motor and non-motor symptoms in patients with PD ≥ 70 (mean: 74.3) years at onset were significantly more severe compared to those in their younger counterparts [21]. The risk of axial symptoms reportedly increases in patients aged ≥ 70 years at PD diagnosis [22]. The discrepancy between previous results and ours may be attributed to the heterogeneity in the study cohorts and methodology.

In this study, the age at diagnosis was slightly older than that in previous studies, in which patients with dementia were excluded.

The prevalence of amyloid positivity (10.0%) in the LOW group (mean age: 64.9 years) was similar to those reported by previous studies among patients with PD without dementia in the same age range (Table 3). Among patients with PD without dementia in their sixties, the prevalence of amyloid positivity is ≤ 10% [17, 18]. Specifically, one study reported a prevalence of 10% in patients with a median age of 61.0 years using the CSFAβ42/Aβ40 ratio [18]. The other study reported a prevalence of 0% in patients with a mean age of 68.8 years using amyloid positron emission tomography (PET) [17] (Table 3).

**Table 3:** Previous reports of the prevalence of amyloid positivity in subjects with normal cognition and PD without dementia.

**Participants**	**Prevalence of amyloid positivity**	**Age (y) of the participants**	**Methods**	**Refs**
In sixties				
Subjects with normal cognition	22.6%	62.5–67.4	CSF Aβ42 or amyloid PET	[25]
Subjects with normal cognition	23.0%	67.9 as mean age	CSF Aβ42 or amyloid PET	[26]
PD without dementia	10.0%	61.0 as median age	CSF Aβ42/Aβ40 ratio	[18]
PD without dementia	0%	68.8 as mean age	Amyloid PET	[17]
This study; PD without dementia	10.0%	64.9 as mean age	CSF Aβ42	
In eighties				
Subjects with normal cognition	42.0%	77.5–82.4	CSF Aβ42 or amyloid PET	[25]
This study; PD without dementia	30.6%	80.2 as mean age	CSF Aβ42	

Subjects with normal cognition = subjects with normal cognition without a diagnosis of Parkinson’s disease, PD = Parkinson’s disease, y = years, Aβ = amyloid-beta, PET = positron emission tomography, Refs = references.

The prevalence of amyloid positivity (30.6 %) in both the LOW and HIGH groups (mean age: 80.2 years) was lower than that in participants with normal cognition in the same age range (Table 3). In participants with normal cognition, the prevalence of amyloid positivity increases with age [23–25] and ranges from 20% [25, 26] to 40% [27] in participants aged approximately 60 and 80 years, respectively, based on evaluations using CSF Aβ42 [25, 26] or amyloid PET [25, 26] (Table 3). The finding that amyloid positivity prevalence was lower in patients with PD without dementia than that in participants with normal cognition in the same age range could be discussed as follows. Although experimental findings are not always consistent and the interactions between Aβ, α-synuclein, and tau remain controversial, several studies have reported interactions between Aβ and either α-synuclein or tau. Hybrid oligomers of Aβ and α-synuclein have been identified in the brains of patients with AD and DLB patients, as well as in amyloid precursor protein/α-synuclein transgenic mice, suggesting a direct interaction between the two [28]. In a mouse model of LBD, Aβ plaques promoted the seeding and propagation of α-synuclein and tau [29], which has been supported by *in vitro* studies showing co-aggregation and direct binding between Aβ and α-synuclein [30, 31]. Based on these findings, our results may be attributed to the shorter preclinical stage of asymptomatic cerebral Aβ deposition in PD [17], with Aβ facilitating the transition from the asymptomatic to dementia stage. Conversely, another study reported that α-synuclein may inhibit Aβ plaque formation [27], which may also explain our findings. Furthermore, given the reported interactions between α-synuclein and tau [32–38], it is plausible that these molecular relationships influence the interaction between Aβ and the other proteins.

Our finding of a significant correlation between CSF t-tau levels and age at diagnosis is consistent with a previous result [21]. This finding can be explained by aging-induced axonal damage in the neurons [39] and age-related co-pathologies, such as primary age-related tauopathy (PART) [40], because advanced age increases the risk of multiple pathologies [41]. For example, a patient with Parkinsonism onset at ≥ 80 years who underwent autopsy after death at 91 years displayed morphological features of PART in addition to PD [16]. Our observation of a significant correlation between t-tau levels and age at diagnosis, regardless of amyloid positivity or negativity (Figure 1F), supports the idea that age-related t-tau elevation is not mainly caused by the common sequential changes reported in AD, such as decreased CSF Aβ42, increased CSF p-tau, and neurodegeneration [42, 43].

A significant correlation between p-tau levels and age at diagnosis in patients with amyloid positivity (Figure 1E) was observed. This may be attributable to tau accumulation induced by α-synuclein [44]. It may also be caused by the increased secretion or reduced clearance of p-tau associated with aging by unrevealed mechanisms in patients with PD with amyloid positivity. Alternatively, it may have been caused by the exclusion of younger patients with high p-tau in the amyloid-positive group from the present study, who may have developed dementia rapidly after amyloid positivization [17].

The AT(N) category distribution of the six patients with MCI and an ApoE ε4 allele in three categories in each group (Supplementary Table 1A) indicates a weak relationship between MCI and amyloid positivity or presence of an ApoE ε4 allele. In this study, we did not reach a conclusion regarding the association between the increased Aβ positivity rate in the HIGH group and clinical manifestations, including MMSE scores.

The underlying pathology of MCI in PD is highly heterogeneous, and this heterogeneity includes different stages of Lewy body pathology, Alzheimer’s disease pathology, and cerebral amyloid angiopathy [45–48]. The pattern of cognitive decline is also reported to be heterogeneous, partly due to variations in neuropsychological assessments used across studies [47], leading to inconsistencies in findings [45–47]. However, impairments in attention and executive function are predominant [49], whereas memory deficits tend to be relatively less frequent [50]. Therefore, further research is needed to elucidate the relationship between the underlying mechanisms of PD-MCI and cognitive decline.

CSF Aβ42 cutoff levels for amyloid positivity vary across the literature, particularly with the INNOTEST® assay (Supplementary Table 2), and prevalence of amyloid positivity varies depending on the cutoff levels (Supplementary Table 3A, 3B). However, our cutoff level is pathology-based, and its validity is supported by the previous studies [51, 52] originating from our institution. In addition, the median (interquartile range) cutoff level among the 30 studies excluding ours in Supplementary Table 2 was 530.2 (452.2–550.0) pg/mL, and three studies adopted the same 500 pg/mL threshold as ours, suggesting that the chosen cutoff aligns with prior studies.

Regardless of the type of biofluid assay or imaging modality used, diagnostic uncertainty exists for values near the cutoff point [53]. When CSF Aβ42 or Aβ42/Aβ40 and amyloid burden measured by amyloid PET are treated as binary data, they show high concordance and are frequently used in clinical practice. However, when analyzed as continuous data, particularly at high levels of amyloid burden, these measures are not interchangeable [54]. Therefore, treating these measures as continuous variables is expected to provide more accurate and deeper insights into the relationships among amyloid accumulation, neuronal loss, and cognitive decline.

The limitations of this study are as follows. First, neuropathological evaluation and CSF α-synuclein data were not available. However, PD was diagnosed according to the diagnostic criteria, and the nigro-striatum impairment by dopamine transporter (DAT) single-photon emission computed tomography (SPECT) was confirmed. We believe that the diagnostic accuracy of PD is reasonable. Second, it has been reported that the Aβ42/40 ratio is better for assessing amyloid positivity [55]; however, data on Aβ40 were not available. Third, all participants were Japanese, resulting in the limited generalizability of the results to other populations [56]. Fourth, healthy controls were not included, but it is unavoidable in retrospective studies because they do not visit medical institutions. Thus, we referred to data of healthy controls in previous studies. Finally, the sample of patients included was relatively small, which restricts the discussion of the differences among the three categories in each group.

To clarify the causal relationship between Aβ positivity and PD progression, a longitudinal, large-scale, multicenter study is needed. It is essential to verify whether Aβ deposition accelerates dementia onset in PD patients in their eighties, as suggested in our study, similarly to those in their sixties. This requires healthy controls, PD with normal cognition, MCI-LB, and PDD/DLB patients. Comprehensive assessments should cover cognition, motor function, and biomarkers—such as CSF or blood-based α-synuclein seed amplification assays [57–59], AD biomarkers [53], and PET imaging including amyloid and tau PET (e.g., ^18^F-MK-6240) [60]. Pathological confirmation, including TDP-43, is also necessary. Ultimately, we must define when, in whom, and which pathological processes to target for disease-modifying therapy to prevent cognitive decline in LBD.

In conclusion, we elucidated the prevalence of amyloid positivity in patients with PD without dementia, whose mean age at diagnosis was 80.2 years, using CSF Aβ42 levels. We believe that our findings will incentivize further studies to identify the best disease-modifying therapy for early PD without dementia.

## MATERIALS AND METHODS

### Participants and clinical protocol

This study was approved by the Institutional Review Board of the Tokyo Metropolitan Institute for Geriatrics and Gerontology. Written informed consent was obtained from all participants or next of kin. We retrospectively evaluated consecutive patients who had been diagnosed with PD based on the UK Parkinson’s Disease Society Brain Bank clinical diagnostic criteria [61] at the Tokyo Metropolitan Institute for Geriatrics and Gerontology between April 2013 and December 2022. The data were accessed from April 28, 2023. Although the authors had access to information that could identify individual participants during or after data collection, all personal information was anonymized and properly protected.

During hospitalization for < 1 week, patients were diagnosed with PD based on clinical history and neurological examination. During the same period, patients underwent examination of cognitive function and orthostatic hypotension (OH), followed by CSF acquisition before administration of dopaminergic agents to confirm the diagnosis.

We included idiopathic PD patients who were diagnosed within 5 years from the onset of a motor symptom, patients with normal cognitive function or MCI [62] (MMSE score ≥ 24), and patients with DAT binding deficit on ^123^I-ioflupane SPECT imaging. Exclusion criteria were dementia; family history of one or more individuals with PD; PD onset at < 40 years; anti-parkinsonian drugs or anti-depressants, such as sulpiride or mirtazapine [63]; and no improvement in motor symptoms after dopaminergic medication. All patients underwent brain magnetic resonance imaging (MRI); patients whose MRI suggested other neurodegenerative diseases, acute or subacute stroke, cerebral amyloid angiopathy [64], or idiopathic normal pressure hydrocephalus were excluded.

Patients were divided into two groups according to age at diagnosis: ≥ 73 or < 73 years old comprised the HIGH and LOW groups, respectively. The justification for 73 as a cut-off age is that the mean age of the older group was approximately 80, the age range we intended to observe, and the numbers of patients in the two groups were relatively balanced at this cut-off, which ensured adequate statistical power.

The following data were investigated: age; disease duration at diagnosis; sex; Hoehn–Yahr scores [65]; presence of motor symptoms such as bradykinesia, rigidity, resting tremor, recurrent falls (> once/year), gait freezing, or festination; presence of at least one non-motor symptom such as sleep disorder (sleep-maintenance insomnia or possibility of rapid eye movement sleep behavior disorder (RBD) evaluated by scores ≥ 5 on the Japanese version of the RBD screening questionnaire [66]); autonomic nervous disorders (constipation, daytime urinary urgency, or symptomatic orthostasis); subjective or objective olfactory disturbance as evaluated using the odor stick identification test for Japanese (scores ≥ 8) [67–69]; psychiatric dysfunction (depression or anxiety); OH defined as a decrease in systolic blood pressure by ≥ 20 mmHg during head-up tilt for 10 min; ^123^I-meta-iodobenzyl-guanidine (MIBG) myocardial scintigraphy positivity; Geriatric Depression Scale scores (≥ 6); education years; MMSE score; ApoE phenotyping; and diabetes mellitus.

To elucidate the AT(N) biomarker profiles of AD in patients with PD, we measured Aβ42, p-tau, and t-tau in CSF. According to the AT(N) biomarker profiles, we counted the number of patients in the three biomarker categories: normal, Alzheimer’s continuum (AD continuum), and non-AD pathologic change in the two groups.

### ^123^I-ioflupane single-photon emission computed tomography assessment

All ^123^I-ioflupane SPECT data were obtained at the Tokyo Metropolitan Institute for Geriatrics and Gerontology. Detailed methods of acquisition of DAT SPECT imaging and calculation of the specific binding ratio (SBR) of the striatal DAT binding using the DAT VIEW software (Nihon Medi-Physics, Co., Ltd, Tokyo, Japan) have been described previously [70]. Positivity was determined based on the mean value of the left and right SBRs < 95% of the lower limit of the prediction interval [71].

### ^123^I-meta-iodobenzyl-guanidine myocardial scintigraphy assessment

All ^123^I-MIBG myocardial scintigraphy data were obtained at the Tokyo Metropolitan Institute for Geriatrics and Gerontology. 111 MBq ^123^I-MIBG (PDR Pharma Co., Ltd, Tokyo, Japan) was injected intravenously, and early and delayed images were obtained with a delay of 15–30 min and 3–4 h, respectively. The heart-to-mediastinum (H/M) ratio and washout rate were calculated as described previously [72]. The respective cutoff values for the widely used H/M ratio and washout rate were 2.20 and 34%, respectively [73, 74]. Positivity was determined based on the presence of at least one of the following: the H/M ratio in the early or delay phase was < 2.20 or the washout rate was > 34%.

### Cerebrospinal fluid analysis

CSF was obtained via lumbar puncture. The first 2 mL of CSF was used for routine examination, and the remaining CSF was directly collected in polypropylene tubes and stored at -30° C until analysis, which was conducted within 2 months.

The CSF concentrations of Aβ42, p-tau, and t-tau were measured using enzyme-linked immunosorbent assay (INNOTEST®, Fujirebio Inc., Gent, Belgium), in accordance with the manufacturer’s protocol, as described previously [52, 75]. The lower detection limit of CSF p-tau was 25.0 pg/mL.

To apply the CSF AD biomarkers to the AT(N) classification [19], we defined the biomarker of Aβ plaques (labeled “A”), fibrillary tau (labeled “T”), and neurodegeneration (labeled “N”) as follows using predetermined institutional cut-off values [75, 76]: A+ indicated Aβ42 < 500 pg/mL, T+ indicated p-tau > 50.0 pg/mL, and N+ indicated t-tau > 300 pg/mL. The Aβ42 cutoff level was pathology-based, and its validity is supported by our previous reports [51, 52].

Categorization in the AD continuum category was indicative of amyloid positivity. To evaluate the correlation between Aβ42, p-tau, or t-tau levels and age at diagnosis, we conducted Pearson’s correlation analysis. To further examine the influence of the amyloid positivity on p-tau or t-tau levels, we examined the relationship between p-tau or t-tau levels and age at diagnosis in patients in two groups based on Aβ42 positivity categorized as “AD continuum” or Aβ42 negativity categorized as “normal” excluding the patients in the “non-AD pathologic change category”.

### Apolipoprotein E phenotyping

ApoE phenotyping was performed using isoelectric focusing, followed by Western blotting.

### Statistics

Contingency table analysis was conducted using Fisher’s exact test. Continuous variables are presented as the mean ± standard deviation (SD) and compared using Welch’s t-test for comparison of means between two groups, or compared using one-way ANOVA for pairwise comparisons of means between two of three groups, followed by *post hoc* analyses using a Tukey’s Honest Significant Difference test. Pearson’s correlation analysis was conducted to determine the relationship between age at diagnosis and other continuous variables. Statistical significance was set at *p* < 0.05. Statistical analyses were conducted using R version 4.1.2 (R Foundation for Statistical Computing, Vienna, Austria) and EZR (Saitama Medical Center, Jichi Medical University, Saitama, Japan) [77]. Missing data were handled using the pairwise deletion approach.

## Supplementary Material

Supplementary Figure 1

Supplementary Tables
