# Supplementary material for: Age-related trends in amyloid positivity in Parkinson’s disease without dementia
Source: Aging (Albany NY). 2025 Aug 6;17(8):2015–32. doi: 10.18632/aging.206297 (PMC12422822; doi:10.18632/aging.206297)
Supplement: Supplementary Figure 1 [file aging-17-8-206297-s001.pdf]

SUPPLEMENTARY FIGURE

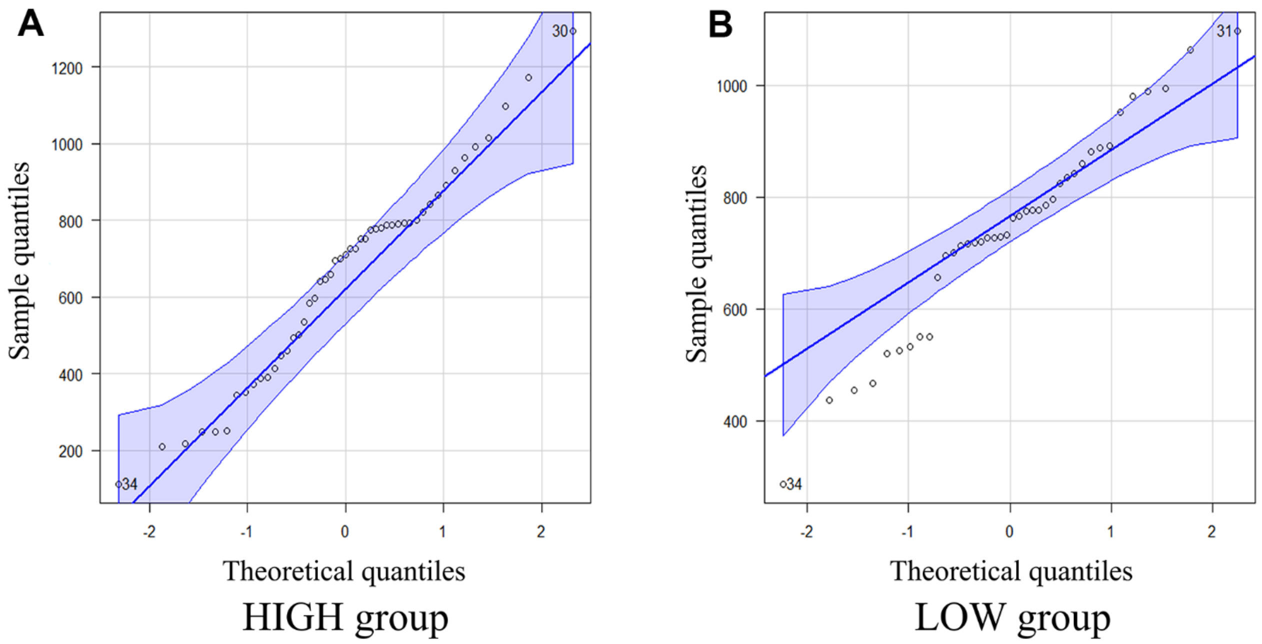

Supplementary Figure 1. Quantile-quantile plots of Aβ42 levels. Quantile-quantile plots of Aβ42 levels in HIGH group (**A**) and LOW group (**B**) showed approximately normal distribution. HIGH group = patients aged ≥ 73 at diagnosis, LOW group = patients aged < 73 at diagnosis, Aβ42 = amyloid-beta 42.
