# Supplementary material for: Age-related trends in amyloid positivity in Parkinson’s disease without dementia
Source: Aging (Albany NY). 2025 Aug 6;17(8):2015–32. doi: 10.18632/aging.206297 (PMC12422822; doi:10.18632/aging.206297)
Supplement: Supplementary Tables [file aging-17-8-206297-s002.pdf]

SUPPLEMENTARY TABLES

Supplementary Table 1A. Distribution of MCI accompanied by clinical data in each AT(N) biomarker category.

AT(N) biomarker categories	Group	Normal	*p* value	AD continuum	*p* value	Non-AD pathologic change	*p* value	Number of missing data		
								Normal	AD continuum	Non-AD pathologic change
Number in each group	HIGH	n = 30		n = 15		n = 4				
	LOW	n = 32		n = 4		n = 4				
Age at diagnosis	HIGH	79.6 (± 3.8)		81.3 (± 5.4)		80.8 (± 3.8)		0	0	0
	LOW	64.7 (± 7.2)		64.3 (± 9.4)		67.0 (± 3.8)		0	0	0
Disease duration (m)	HIGH	21.1 (± 14.1)		15.2 (± 13.1)		6.5 (± 3.5)		0	0	0
	LOW	23.6 (± 14.8)		13.3 (± 13.2)		7.8 (± 6.0)		0	0	0
Education (y)	HIGH	11.6 (± 2.7)	0.001**	12.1 (± 2.7)	0.48	11.5 (± 4.5)	0.81	7	4	2
	LOW	14.5 (± 2.7)		13.3 (± 1.9)		13.0 (± 5.3)		11	1	0
MMSE scores	HIGH	26.8 (± 2.0)	0.003**	27.6 (± 1.9)	0.86	27.7 (± 1.2)	0.96	4	3	1
	LOW	28.4(± 1.7)		27.3 (± 1.7)		27.8 (± 2.3)		7	1	0
MCI, n (%)	HIGH	3 (10.0%)		1 (6.7%)		0		0	0	0
	LOW	1 (3.1%)		0		1 (25.0%)		0	0	0
ApoE ε4, n (%)	HIGH	4 (13.8%)		6 (42.9%)		0		1	1	1
	LOW	4 (14.2%)		1 (25.0%)		0		4	0	1

Continuous variables are presented as the mean (± standard deviation). AD continuum = Alzheimer's continuum, Non-AD pathologic change =Non-Alzheimer's pathologic change, HIGH = a group of patients aged ≥ 73 at diagnosis, LOW = a group of patients aged < 73 at diagnosis, MMSE = mini-mental state examination, MCI = mild cognitive impairment, ApoE = Apolipoprotein E, Aβ42 = amyloid-beta 42, p-tau = phosphorylated tau, t-tau = total tau, ** = $p < 0.01$. *P* values represent the result of Welch's t test comparing the HIGH group vs. the LOW group within each AT(N) category.

Supplementary Table 1B. Contingency table for Fisher's exact test showing the numbers of MCI in each AT(N) category within the HIGH group.

	Normal	AD continuum	Non-AD pathologic change	Total
MCI	3	1	0	4
Normal cognition	27	14	4	45
Total	30	15	4	49

MCI = mild cognitive impairment, HIGH group = a group of patients aged ≥ 73 at diagnosis, AD continuum = Alzheimer's continuum, Non-AD pathologic change = Non-Alzheimer's pathologic change.

Supplementary Table 1C. Contingency table for Fisher's exact test showing the numbers of MCI in each AT(N) category within the LOW group.

	Normal	AD continuum	Non-AD pathologic change	Total
MCI	1	0	1	2
Normal cognition	31	4	3	38
Total	32	4	4	40

MCI = mild cognitive impairment, LOW group = a group of patients aged < 73 at diagnosis, AD continuum = Alzheimer's continuum, Non-AD pathologic change = Non-Alzheimer's pathologic change.

Supplementary Table 2. Cutoff levels of CSF amyloid-beta 42 in prior studies using INNOTEST® and our study.

No.	CSF Aβ42 cutoff (pg/mL)	Study	References
1	333	J-ADNI	26
2	380	Krakow	25
3	430	Brussels	25
4	445	Lisbon	25
5	450	Bremen	25
6	450	Gothenburg	25
7	450	Ljubljana	25
8	450	Mannheim	25
9	450	Thessaloniki	25
10	459	St. Louis	25
11	482	Mattsson	25
12	490	Athens	25
13	500	Barcelona CUH	25
14	500	Brescia	25
15	500	Nijmegen	25
16	500		This study
17	542	Coimbra	25
18	550	Amsterdam	25
19	550	Barcelona HSP	25
20	550	DESCRIPA	25
21	550	Lausanne	25
22	550	LeARN	25
23	550	Lorenskog	25
24	550	Madrid	25
25	550	Perugia	25
26	550	Stockholm	25
27	600	DCN	25
28	600	Scinawa	25
29	610	Warsaw	25
30	638.5	Antwerp	25
31	662.65	Chandigarh	25

Median (IQR) cutoff level: 530.3 (452.3-550.0)
Of the 31 cutoff levels, 30 were taken from prior studies cited in Table 3 (29 from Reference [25] and one from Reference [26]), and the remaining one was our own. These levels ranged from 333 to 662.65 pg/mL. CSF = cerebrospinal fluid, No.= Number, Aβ42 = amyloid-beta 42, J-ADNI = Japanese Alzheimer's Disease Neuroimaging Initiative, IQR = interquartile range. See the original paper about study names.

Supplementary Table 3A. Comparison of the prevalence of amyloid positivity between the two groups at a cutoff level of 400 pg/mL.

	HIGH group (N = 49)	LOW group (N = 40)	Odds ratio	95% CI	*p* value
Amyloid positivity, n (%)	11 (22.4%)	1 (2.5%)	11.1	1.5–496.9	0.01**

HIGH group = patients aged ≥ 73 at diagnosis, LOW group = patients aged < 73 at diagnosis, CI = confidence interval, ** = $p < 0.01$. *P* value represents the result of Fisher's exact test.

Supplementary Table 3B. Comparison of the prevalence of amyloid positivity between the two groups at a cutoff level of 600 pg/mL.

	HIGH group (N = 49)	LOW group (N = 40)	Odds ratio	95% CI	*p* value
Amyloid positivity, n (%)	19 (38.8%)	9 (22.5%)	2.2	0.8–6.3	0.11

HIGH group = patients aged ≥ 73 at diagnosis, LOW group = patients aged < 73 at diagnosis, CI = confidence interval. *P* value represents the result of Fisher's exact test.
